# Service Innovation in Human Resource Management During COVID-19: A Study to Enhance Employee Loyalty Using Intrinsic Rewards

**DOI:** 10.3389/fpsyg.2021.627659

**Published:** 2021-02-25

**Authors:** Muhammad Ibrahim Abdullah, Dechun Huang, Muddassar Sarfraz, Muhammad Waqas Sadiq

**Affiliations:** ^1^Business School, Hohai University, Nanjing, China; ^2^Department of Management Sciences, Comsats University Islamabad, Lahore, Pakistan; ^3^Binjiang College, Nanjing University of Information Science and Technology, Wuxi, China; ^4^Department of Management Sciences, Comsats University Islamabad, Sahiwal, Pakistan

**Keywords:** job satisfaction, employee loyalty, nurses, service innovation, COVID-19, human resource management

## Abstract

This research focuses on the employee loyalty aspect of private hospitals in Pakistan during the COVID-19 pandemic, seriously impacted by strict work demand and work-family conflict. To manage this issue, social rewards and psychological rewards played a role as a mediator. The study uses a causal research design with a correlational study design in a non-contrived environment. Minimal researcher interference has been assured. AMOS 24 has been used to deal with the mediation in study design with bootstrap methodology. The study was conducted on 250 nurses of different private hospitals across Punjab province using a proportionate stratified sampling technique. A finding of this study suggests that nurses remain loyal to their organizations despite having uncompromising work demands and facing work-family conflict when they are provided with social and psychological rewards on their job by their organizations.

## Introduction

The health care sector is considered one of the crucial sectors of all. Its primary purpose is to provide top-class facilities and assistance to the concerned persons. Suppose the health care industry's mission and purpose are to provide top class assistance (Peltier et al., 2003). In that case, the professionals who work for this sector must be considered the central point for its effective running. Further, the well-being of the professionals will ensure the availability of high standards in this sector.

Hospitals need skilled staff, especially nurses, to achieve organizational effectiveness and provide outstanding patient care. It is tough to locate successful nurses, considering the ongoing global nursing crisis (Price and Mueller, 1981). The same situation goes all over Pakistan, especially in one of the biggest Punjab province.

Nurses could be considered one of the essential parts of the health care industry. Their attentive and vigilant services are incredibly vital for patient's treatment and recovery. That could only be possible if they are satisfied with their job and showed loyalty toward their current organization (Abdullah et al., 2020; Kengatharan and Kunatilakam, 2020). Hospital administration needs to provide the best working conditions for the professionals who work there, so that patient's health and treatment must not be compromised at any level. While the COVID-19 situation is at its peak at the start of the year 2020, competent and professional medical staff is uncompromised in this situation. Hospitals, especially those that belong to the private sector, must develop innovative HRM practices to retain their medical team, including nurses (Needleman and Hassmiller, 2009).

This study aims to provide insight into nurses' minds from a work-family conflict and work demand point of view. While especially considering the COVID-19 situation as a significant influencer over society. Fulfillment of this study's objectives will ultimately provide reliable information to the hospitals and health organizations to keep nurses loyal to their organization while considering only the intrinsic or non-monetary rewards. Punjab province is one of the main hubs for the best medical care and facilities all over Pakistan, and it is filled with numerous private hospitals. During the COVID-19 situation, the economic conditions declined sharply worldwide, and Pakistan is no exception in this scenario. This latest pandemic brings many managerial and organizational problems due to a sudden increase in the patient count and work-family conflict situation, which arises in every house with the evolving pandemic situation. Every house member of these nurses is worried about their loved ones, forcing them to quit their job during this daunting situation.

So, hospitals are looking forward to non-intrinsic rewards to cope with this work and family conflict situation. Due to declining economic conditions, they cannot afford monetary rewards on a more extensive and more prolonged scale. So, they have to focus on the management of non-monetary benefits. This study focuses on finding viable solutions for hospital management so they could be able to retain their valuable employees with the help of non-monetary rewards during this COVID-19 situation.

### Study Significance

Every organization wants to apply a combination of monetary and non-monetary rewards so they could be able to retain their valuable employees in the longer run. This study will help hospital administration promote a healthy work environment to maintain their existing staff and attract competent and capable nurses. Further, by promoting a healthy work environment, employee loyalty will ultimately increase, resulting in nurses' more efficient and effective performance. This study will also be helpful full of expanding employee loyalty toward that organization. This study's findings will also help the administration of other public and private hospitals in Pakistan to enhance their environment and working standards.

## Literature Review and Study Background

It is paramount to consider why the study is carried out on these variables before the comprehensive literature review. It is essential to investigate these variables before conducting a survey. We need to know why these variables are chosen as independent, mediator, and dependent variables. What kinds of hypotheses endorse them in their entirety? As discussed earlier, due to COVID-19, the environment is evolving swiftly, and health organizations must bring creativity to their human capital framework. This pandemic situation will ultimately help sustain its critical workers (Hafeez et al., 2020).

**Figure 1:**
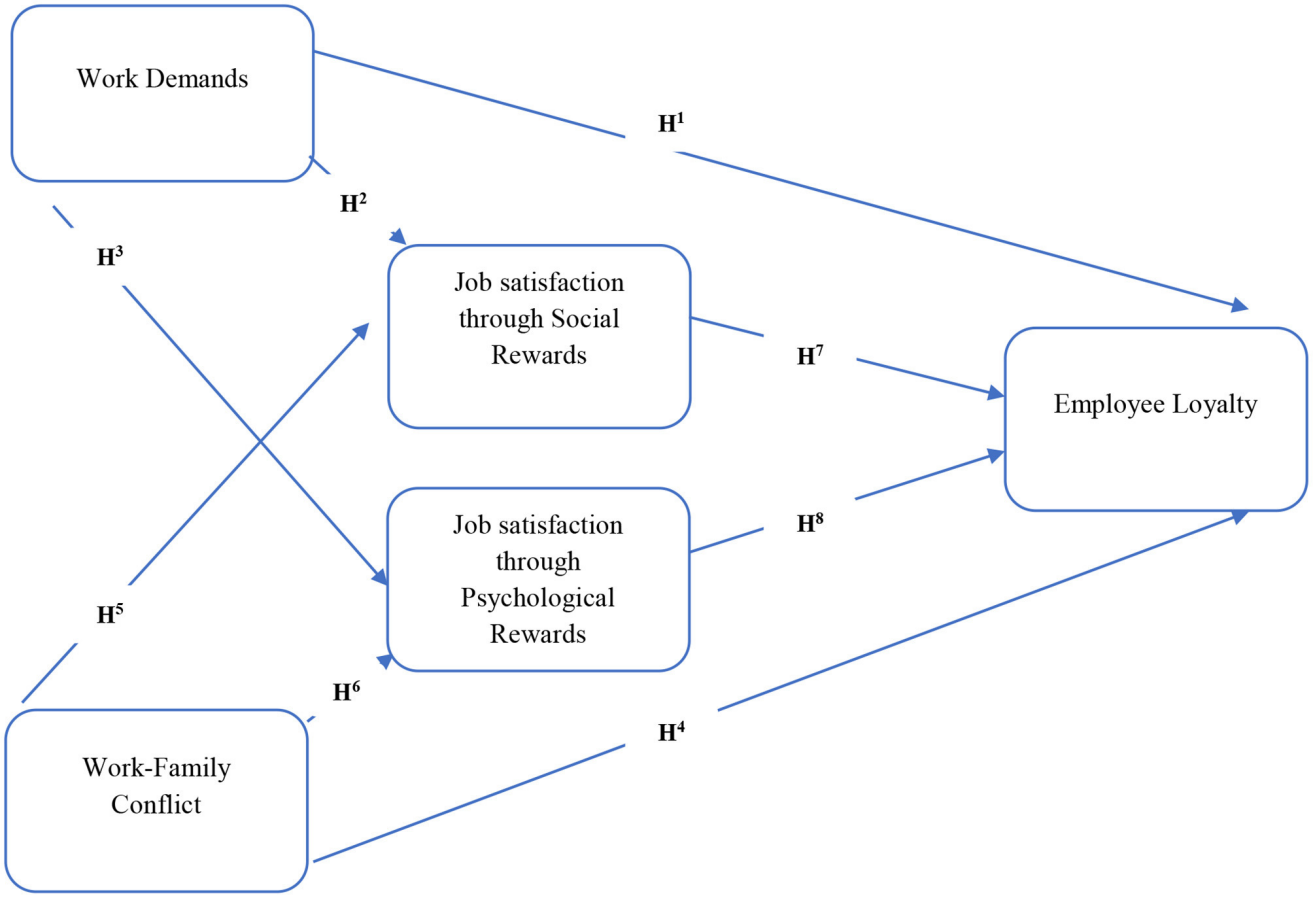
Theoretical model.

COVID-19 also brings the work-family conflict factor to a new level. Everyone is concerned about the health of their loved ones who are working in hospitals and acting as a front-line soldier before this deadly virus. Their family members ask them to leave their jobs or minimize contact with their potential COVID-19 patients. In this scenario, hospitals need to bring incentives to uplift their health staff's social and psychological aspects. So, they could be willing to perform their services during this too tense scenario. After considering this scenario, this study comes up with the following theoretical model, which is also theoretically supported by the boundary theory and border theory presented by Lavassani and Movahedi (2014). This theory discusses the impact of work-family conflict on employee loyalty toward organizations and their job satisfaction Figure 1.

Further, previous literature also supports this kind of study (Benligiray and Sönmez, 2012; Tziner and Sharoni, 2014; Yang et al., 2017) discusses that health organizations need to work on their employees management skills. So they could be able to understand the behavior of their employees more comprehensively, which will result in a more enhanced loyalty toward the organization and job satisfaction. This research gains more importance due to the COVID-19 scenario because health organizations now need to raise their employee's loyalty. Cognitive CEO enhances environmental responsibility (Sarfraz et al., 2020). Perceived organizational states that employees are valued by the organization (Sarfraz et al., 2019).

### Work Demand

In the healthcare industry, the demand for nurses' work is concerned with the outcome of nurses. It can but is not restricted to receiving patients or their relatives' grievances for the inability to fulfill their demands. Patients or their families had a wrong impression. That may also entail not getting enough time to satisfy patients and family members (AbuAlRub, 2004). Patients could often be unaware of the degree to which patients' symptoms or procedures reveal them. They cannot attend to patients through excessive tasks in their office. Moreover, they must maintain professional units other than their own (Ernst et al., 2004). By considering and discussing the information mentioned above, the below-mentioned hypothesis is proposed for further analysis.

All the below-mentioned hypotheses are supported by the boundary theory (Lavassani and Movahedi, 2014). These hypotheses follow this theory's parameters, which discuss the connection between an employee's personal and work life from different significantly essential perspectives.

H1: Work demands create a positive impact on employee's loyalty toward their organization.

H2: Work demands mediated by Social rewards positively impact employee's loyalty toward their organization.

H3: Work demands mediated by Psychological rewards positively impact employee's loyalty toward their organization.

### Work-Family Conflict

The work-family dispute is called a particular type of inter-role rivalry where work-family roles are conflicting. Work-family conflict has two dimensions: work-to-family conflict (WFC) happens when work-to-family interactions and responsibilities intervene. The conflict between family and employment (FWC) occurs as family commitments conflict with work-life (Drexler and Fujimoto, 2008). Jobs and family clash when a career interfere with individuals, other professional positions, and desires. They will differ from time to time for mates, fitness, military service, schooling (Grzywacz et al., 2006). Work and family conflicts are a type of inter-role conflict where one area sometimes impacts or spills into other domains.

That means that the work domain is used to influence or spill the family domain, and the family domain influences or spills the work domain (Ghislieri et al., 2017). When efforts to fulfill work, role demands interfere with one's ability to meet family demands and vice versa. It also occurs when one role interferes with an individual's effectiveness in other parts, and these roles are work roles and family roles (Thomas and Ganster, 1995). It is further defined as: “work-family conflict refers to the degree to which the demands of one role has an impact on the demand of the other role.” It's a conflict as a degree to which an individual obligation to fulfill family requirements is affected by the work-life interruption and a degree to which a personal responsibility to achieve job requirements are affected by family (AlAzzam et al., 2017). The following presented hypothesis is related to the discussion mentioned above and encompasses this research study's philosophical approach.

H4: Work-family conflict creates a positive impact on employee's loyalty toward their organization.

H5: Work-family conflicts mediated by Social rewards positively impact employee's loyalty toward their organization.

H6: Work-family conflicts mediated by Psychological rewards positively impact employees' loyalty toward their organization.

### Job Satisfaction Through Social Rewards

An evaluation of the literature illustrates that job satisfaction phenomena have been broadly studied in many fields, including military, nursing, public sector, business, psychology, and sociology. The idea has developed over time. But did not produce a distinctive implication. It is a composite phenomenon influenced by various factors (Ravari et al., 2012). We could define job satisfaction as a “pleasurable or positive emotional state resulting from the appraisal of one's job or job experiences” (Locke, 1976). Broadly we could relate job satisfaction as “the extent to which employees like their jobs” (Stamps, 1997).

Social rewards play a dominant role in developing and maintaining employees' satisfaction regarding their jobs across all occupational groups. Extrinsic rewards given in an organization appear to play a significant role regarding this matter (Mottaz, 1985). Social rewards could be considered as a vital element for better employee productivity (Roy, 1953). Young women and female workers give more importance to social rewards than rewards related to job leisure-related aspects(Marini et al., 1996). The social rewards are used loosely across studies and typically denote any social stimuli or interactions that participants experience as rewarding (Beauchamp et al., 1983). Social rewards propel individuals into social proximity in the wild under more expensive and patchier conditions than the laboratory cage, motivating communication with others (Izuma et al., 2008). Social benefits create a good sense of well-being as one experiences belonging is recognized during social activities by others, and can experience superiority through tasks and social interactions (Ocean and Smith, 1993). Social rewards are more cheap or free and can be even more potent than materials. One can be given even more often and immediately after behaviors you like (Panksepp, 2000).

The social reward is a loyalty-Marketing program that rewards users for tweeting and posting fakebooks about the brand they like and working with many casinos. Social rewards engage similar neural circuits as non-social and alter neural sensitivity to the value of non-social rewards in adolescence, precisely when another person presents. At the same time, participants engage in a risk-taking process (Jessen, 2010). The following proposed hypothesis is related to the discussion mentioned above and encompasses this research study's philosophical approach.

H7: Social rewards create a positive impact on employee's loyalty toward their organization.

### Job Satisfaction Through Psychological Rewards

Job satisfaction is “an attitudinal variable that represents the extent to which people like (satisfaction) or dislike (dissatisfaction) their jobs” (Spector, 1997). We could consider job satisfaction as one of the numerous constructs that have frequently been used to explain the nursing staff's functioning circumstances, mainly due to its significant associations with other variables (Cortese, 2010). From the 19th century onwards, job satisfaction has been studied extensively due to its critical nature and relationship with other important variables related to organization and employee psychology. Psychological rewards given to employees shows a strong relationship between work and family life balance, thus enhancing the satisfaction about one's job and increasing the trust toward their organization (Voydanoff, 2004).

Psychological rewards, in which we could consider admiration, high opinion, and essential work, are characteristic of personality improvement, ultimately enhancing a sense of worth and satisfaction (Behson, 2002). Psychological rewards such as status enhancement and personality enrichment will also play a vital role in improving employee productivity and loyalty and trust in its organization (Allen, 2001). A reward is an appetizing stimulus provided to alter the actions of a person or any other species. Rewards act as a strengthener. Rewards are an analytical way to characterize an entity's beneficial meaning, activities, or an inner physical condition that a person assigns (Hofmans et al., 2013). Operational conditioning, also named instrumental training, is a form of learning, which happens by incentives and consequences for actions. Via functional operation, a person connects a specific activity with a result (Scott-Ladd et al., 2006).

According to the pragmatic approach to belief, knowledge is commonly viewed as a justified and true belief and is more rewarding than satisfying social and material needs. A reward is a subjectively pleasurable or satisfying event or experience that follows the completion of a task. The attainment of reward is a reinforcement for the conditioning paradigm preceding action (De Gieter et al., 2008).

Behaviors are committed to strengthen or prevent punishment. Incentive theory suggests that acts are targeted at receiving incentives. The word incentive describes an occurrence creating an excellent or optimistic emotional experience. The incentive is also used to convey an event that raises the likelihood or rate of action-related activity (Jehanzeb et al., 2012). The following presented hypothesis is related to the discussion, as mentioned earlier, and encompasses this research study's philosophical approach.

H8: Psychological rewards create a positive impact on employee's loyalty toward their organization.

### Employee Loyalty

These days, employee loyalty is vital for any company and organization to enhance and improve their organizational structure, excellence, and efficiency. Now-day, employee loyalties are declining toward their company or organization. Several organizations want to uncover some methods to enhance their employee loyalty. If an organization understands its employee's needs effectively and efficiently, this phenomenon could cultivate employee loyalty toward its organization (Vischer, 2005).

We could define Employee loyalty as employees consider that they have no doubts or qualms regarding working for their company or organization. They believe that it will be the best choice for them in the present and future. From an organization's perspective, an employee will be considered an important resource and vital asset. Different benefits and rewards can increase the employee's productivity and loyalty toward the organization. There is a high cost for changing an employee regardless of his or her position in an organization. Because building organizational commitment is not an easy job. Employers need to realize why their employees are psychologically associated with their organization—most of the time, it is much more than regular perks and benefits. Psychologically connected and loyal employees are the best employees because they are engaged and creative, and they feel corroborated and respected (LaMalfa, 1996).

## Research Methodology

A hypothesis study has been used for this research to explain the nature of the relationship between understudy variables. The data as shown Table 1 data in of nurses have been drawn from the concerned HR offices of different private hospitals. The reason for selecting private hospitals is that they do not offer permanent job structures like a public or government-owned hospital. That is why they need to focus more on their work environment and reward system. So, they could be able to retain their valuable staff in the longer run.

**Table 1:** Demographic profile of the respondents.

**Category**	**Subdivision**	**Frequency**	**Percentage**
Marital status	Married	150	60
	Un-Married	100	40
Currently working ward	Medicine	28	11.2
	Gynecology	28	11.2
	Surgery	28	11.2
	Pediatrics	28	11.2
	ENT	28	11.2
	EYE	28	11.2
	Orthopedics	27	10.8
	Cardiology	27	10.8
	Physiotherapy	28	11.2
Age	Below 25 years	40	16
	25–30	65	26
	31–35	80	32
	36–40	45	18
	40 and above	20	8
Education	Intermediate	110	44
	Bachelors	128	51
	Masters	12	5
	M.Phil	0	0
	Phd	0	0
Experience	Below 5 years	50	20
	6–10 years	85	34
	11–15 years	65	26
	16–20 years	40	16
	Above 20 years	10	4

This study has used a Correlational type of investigation because it needs to check the variables' relationship through hypotheses. Research has been conducted in a natural environment. That is why it will be considered a non-contrived study setting. This study has minimal researcher interference toward respondents regarding the filling up of questionnaires. In this study, the data is collected from nurses; that's why this study's unit of analysis is individual. The researcher has implemented a cross-sectional study method for this study. It involves the study of all a population, or a representative subset, at one specific point in time.

The stratified sampling methodology is used to collect an equal number of nurses from each ward. Because of the risk of infection from the COVID-19 virus is the same in every ward, it is vital to collect data through stratifying sampling methodology. Nurses are the leading group in the healthcare sector.

The nurse's attendance register is the population framework in this study, through which the data is collected for this research study. In some hospitals, the data was collected by the researcher. In contrast, it was collected with different friends' help to connect with that hospital medical staff and other doctors in some hospitals. In some cases, the data is collected from the homes of nurses who are working in different private hospitals.

### Empirical Settings and Data Collection

These studies have been conducted on nurses of different private hospitals of Punjab province, 500 questionnaires have been distributed among them, and almost 270 questionnaires have been received, maintaining the response rate of 55%. A reliable and valid questionnaire has been used for this study. Among which 20 questionnaires were with incomplete information, so the analysis was done with 250 complete responses. Respondents' demographic profile is mentioned below.

The respondents were nurses of different hospitals, and the data collected during the pandemic situation by using various resources to avoid human contact as applicable as possible. All private hospitals have different departments like Surgery, Orthopedics, Pediatrics, Cardiology, Ear, nose, and throat (ENT), Gynecology. So, we will use the Stratified Sampling method. In this way, we could be able to draw a sample of nurses from each department.

### Study Instrument

For measuring Work demand/Job Stress and work-family conflict, we will use the “Nurses' Occupational Stressor Scale,” which is specifically developed for nurses by Chen et al. (2020). This scale is based on a five-point Likert scale. For social and psychological rewards, this study will use the Mueller-McCloskey Satisfaction Scale, commonly known as (MMSS), which is developed in (1990). It was specially designed to investigate the level of job satisfaction amongst the nurses. It was a multidimensional survey developed to evaluate and examine nursing team satisfaction (Mueller and McCloskey, 1990). The instruments were rated and measured on a 5-point Likert scale with higher numerical values showing greater satisfaction. The reliability, construct validity of the scale, and the internal consistency (Cronbach's alpha = 0.89) were acceptable (Misener et al., 1996). While investigating employee loyalty, we will use a proven questionnaire known as the employee loyalty acid test, which is used to measure employees' loyalty toward their organization (Subramani, 2013).

## Study Results

### Confirmatory Factor Analysis

It is necessary to conduct the confirmatory factor analysis for accurate and precise results for all variables. For this study, it is decided to conduct a pooled CFA analysis. It runs all the latent variables at the same time to achieve the required model fitness. The pooled CFA method is a lot easier and better than the Individual CFA since it runs all the latent variables simultaneously, which is time-saving (Afthanorhan et al., 2014; Chong et al., 2014).

The model fit indices shown in Table 2 acceptable fit between the data and the proposed measurement model. The values of the Comparative Fit Index (CFI = 0.938), Root Mean Error of Approximation (RMSEA = 0.049). Chi-square to Degree of Freedom Ratio (x^2^/df = 1.590) are all meeting the cut-off criteria, so the values of the fitness indices meet the excellent standards for model fitness (Lomax and Schumacker, 2004; Hoe, 2008; Anderson et al., 2010).

**Table 2:** Pooled CFA model fitness tests.

**Name of Category**	**Name of index**	**Index full name**	**Value in analysis**	**Acceptable value**	**References**
Absolute Fit	RMSEA	Root mean square of error approximation	0.049	<0.80	Browne and Cudeck, 1993
Incremental Fit	CFI	Comparative fit index	0.938	>0.90	Bentler, 1990
Parsimonious Fit	Chisq/df	Chi square/degrees of freedom	1.590	>5	Hu and Bentler, 1999

Pooled CFA Model Fitness Tests after running the pooled CFA, it is also necessary to check and verify each item's reliability for further research. CFA of this study's the data was used to measure reliability as shown in Table 3, convergent validity, and discriminant validity. The data was used to measure discriminant validity as shown in Table 4. The reliability of the measurement scales was measured with composite reliability, which is preferred to report a scale's reliability (Netemeyer et al., 2003), a widely used indicator.

**Table 3:** Pooled confirmatory factor analysis (independent, mediating & dependent variable).

**Scale**	**Items**	**Factor loadings**	**Scale reliability**
Work Demand	I am worried about receiving complaints from patients or their relatives for not meeting their demands.	0.739	0.716
	I have to bear the negative sentiment of patients or their relatives.	0.640	
	I do not have sufficient time to meet patients' and their relatives' demands.	0.656	
	I am unsure of the extent of patients' conditions or treatments that I should reveal to them.	0.742	
	Excessive duties in the workplace prevent me from attending to patients.	0.705	
	I have to maintain professional units other than my own.	0.816	
Work-family conflict	The burden of work affects my domestic life.	0.770	0.777
	The amount of time my job occupies makes it difficult for me to fulfill family responsibilities.	0.993	
	The burden of work makes it difficult for me to undertake my personal chores and/or engage in hobbies.	0.558	
	My job produces a strain that makes it difficult for me to fulfill my family duties.	0.856	
	I have to adapt my schedule for family activities/outings to accommodate my work responsibilities.	0.708	
Job satisfaction through social rewards	Your nursing colleagues are cooperative with you during your job.	0.707	0.703
	Physicians you work with are cooperative with you during your job.	0.739	
	The hospital provides opportunities for social contact at work.	0.640	
	The hospital provides opportunities for social contact with your colleagues after work.	0.701	
	Hospital administration provides opportunities to interact with other disciplines.	0.776	
	Hospital administration provides opportunities to interact with faculty.	0.656	
Job satisfaction through psychological rewards	Hospital administration provides opportunities to belong to department and institutional committees.	0.742	0.716
	Hospital administration provides opportunities to participate in nursing research.	0.802	
	Hospital administration provides opportunities to write and publish.	0.816	
	You were admired by your immediate supervisor.	0.605	
	You have received recognition for your work from superiors.	0.775	
	You have received recognition for your work from peers.	0.825	
	You have received a fair amount of encouragement and positive feedback on your work.	0.763	
	You have given control over your work setting.	0.543	
	You have given opportunities for career advancement.	0.705	
	You have a given amount of responsibility.	0.816	
	You have given control of your work conditions.	0.643	
	You have given a chance to participate in organizational decision-making.	0.641	
	I would like to be working at this organization 2 years from now.	0.634	
Employee loyalty with the organization	Hospital administration communicates openly and honestly	0.825	0.719
	Hospital administration is committed to win/win solutions (does not take advantage of its staff or patients)	0.763	
	I trust the Hospital administration to behave with fairness and integrity	0.543	
	Employee loyalty is appropriately valued and rewarded at this organization	0.705	
	I believe this hospital deserves my loyalty	0.816	
	Over the past year, my loyalty to this hospital has grown stronger	0.643	
	This Hospital administration values people and relationships ahead of short-term benefits	0.641	
	This hospital sets the standard for excellence in its industry	0.816	

**Table 4:** HTMT analysis.

	**Work demand**	**Work-family conflict**	**Social rewards**	**Psychological rewards**	**Employee loyalty**
Work demand					
Work-family conflict	0.075				
Social rewards	0.172	0.067			
Psychological rewards	0.117	0.090	0.048		
Employee loyalty	0.220	0.030	0.035	0.568	

Discriminant validity is used to confirm that the measurement scales are distinct from other measures used in the study. Discriminant validity was measured using the HTMT analysis in which the cut-off criteria for strict discriminant validity are 0.850 and for liberal discriminant validity is 0.900 (Henseler et al., 2015). Therefore, it is established that all the measurement scales used in the study differ from each other, so the data used in our study fulfills the requirements of convergent and discriminant validity and is suitable for further analysis.

### Structural Equation Modeling

Structural equation modeling (SEM) was used in the Structural model to test the hypotheses, using AMOS 24. As the proposed model contains mediation, the SEM technique was used to analyze all of the paths simultaneously (Iacobucci et al., 2007; Hoe, 2008; Alavifar et al., 2012). The model fit indices as shown in Table 5 for the structural model are meeting the acceptance criteria.

**Table 5:** SEM, model fitness tests.

**Name of category**	**Name of index**	**Index full name**	**Value in analysis**	**Acceptable value**	**References**
Absolute fit	RMSEA	Root mean square of error approximation	0.057	<0.80	Browne and Cudeck, 1993
Incremental fit	CFI	Comparative fit index	0.914	>0.90	Bentler, 1990
Parsimonious fit	Chisq/df	Chi square/degrees of freedom	1.814	>5	Hu and Bentler, 1999

### Hypothesis Testing

The results of the structural model are shown in Tables 6, 7.

**Table 6:** Structural model: direct effects.

**Hypothesis**	**Causal path**	**Lower bound**	**Upper bound**	***P*-value**	**Standardized estimated**
H1	Work demand → Employee loyalty	−0.162	0.093	0.690	−0.032
H4	Work-family conflict → Employee loyalty	−0.183	0.026	0.206	−0.080
H7	Social rewards → Employee loyalty	0.096	0.378	0.008	0.335
H8	Psychological rewards → Employee loyalty	0.219	0.464	0.001	0.430

**Table 7:** Results of structural model: indirect effects.

**Hypothesis**	**Causal path**	**Lower bound**	**Upper bound**	***P*-value**	**Standardized estimated**
H2	Work demand → Social rewards → Employee loyalty	0.060	0.174	0.001	0.20
H3	Work demand → Psychological rewards → Employee loyalty	0.027	0.140	0.006	0.35
H5	Work-family conflict → Social rewards → Employee loyalty	0.052	0.153	0.035	0.15
H6	Work-family conflict → Psychological rewards → Employee loyalty	0.019	0.098	0.004	0.12

The SEM statistics show that H1 (Work DemandEmployee Loyalty), H4 (Work-family conflictEmployee Loyalty) are rejected on the grounds of significance level, as the SEM results show that the *P*-values of these hypotheses are not significant. These results suggest that these variables do not have a direct significant positive impact on employee loyalty. While H7 (Social RewardsEmployee Loyalty), H8 (Psychological RewardsEmployee Loyalty) are accepted on the grounds of significance level, as the SEM results show that the *P*-values of these hypotheses are significant. These results suggest that these variables have a direct significant positive impact on employee loyalty.

These results showed the complete picture of this research study. The study showed that H2 (Work DemandSocial RewardsEmployee Loyalty, β = 0.20, *P* = 0.001) is positively significant and suggests that when organizations provide social rewards to their valuable employees, their loyalty remains firmly with the organizations. Even when the work demand or job stress is exceptionally high, we could see that during this current scenario based upon the COVID-19 pandemic, the hospitals are under immense pressure during this present scenario.

The study showed that H3a (Work DemandPsychological RewardsEmployee Loyalty, β = 0.35, *P* = 0.006) is also positively significant and suggests that organizations provide a psychological reward to their valuable employees, their loyalty remains firmly with the organizations. Even when the work demand or job stress is exceptionally high, we could see that during this current scenario based upon the COVID-19 pandemic, the hospitals are under immense pressure during this present scenario.

This hypothesis showed that H2b (Work-Family ConflictSocial RewardsEmployee Loyalty, β = −0.15, *P* = 0.035) is positively significant and suggests that organizations provide social rewards to their valuable employees, their loyalty remains firmly with the organizations. Even when the work demand or job stress is exceptionally high, we could see that during this current scenario based upon the COVID-19 pandemic, the hospitals are under immense pressure during this present scenario.

This specific hypothesis showed that H3b (Work-Family Conflict Psychological RewardsEmployee Loyalty, β = 0.12, *P* = 0.004) is also positively significant and suggests that organizations' psychological rewards valuable employs then their loyalty remains firmly with the organizations. Even when the work demand or job stress is exceptionally high, we could see that during this current scenario, which is based upon the COVID-19 pandemic, the hospitals are under immense pressure during this current scenario.

## Discussion

It is evident from the findings that nurses' job satisfaction in hospitals could be ensured through social and psychological rewards while considering work demand and work-family conflict as an independent variable while considering the COVID-19 pandemic situation as a huge influencer. Even though previous studies don't have COVID-19 conditions, they still support such results (Lu et al., 2005; Dignani and Toccaceli, 2013). Another study done by Ma et al. (2003) suggests that intrinsic rewards played a vital role in changing employee behavior toward their organization. They remain loyal to their organizations, and the low turnover ratio ensures they have a high level of employee performance, leading to patient satisfaction.

While looking into the results, we could find that in the case of the work demand variable, the psychological rewards could play a vital role in enhancing nurses' loyalty toward their organization compared to the social rewards (Rice et al., 2017). Suppose any hospital administration wants to invest resources in developing these kinds of rewards and are looking forward to eradicating the negative impact of work demand or job stress on employee loyalty toward their organization if they don't have enough resources to develop both kinds of rewards. In that case, they must need to focus on developing psychological rewards. If they want to work on work-family conflict, they should focus on developing social rewards.

## Conclusion

It is concluded from the above discussion and findings that nurses are one of the essential pillars in the health sector. Without them, it is impossible to run the hospital and different health organizations. That is why it is also imperative to keep them happy and loyal to their organization to work more in the same organization (Bakertzis and Myloni, 2020), thus generating more productivity and successful work. To complete that kind of task, we must focus on the social and psychological rewards. According to this study findings, they will help the organizations keep their employee loyal and happy with them. Finding new avenues for building employee loyalty is not a new topic in literature. The researchers could also introduce online HRM services to enhance the impact of social and psychological rewards (Sadiq et al., 2020a,b).

### Study Managerial Implications

This study could also be used to investigate other industry employee behavior, including education, banking, aviation, etc. Still, they must use other questionnaires because the questionnaire used to collect nurses' data in this study is specifically designed for nurses. They could also adapt this questionnaire according to their requirements after changing its statement and applying CFA for reliability and validity. Moreover, this study could also be conducted in other geographical scenarios to verify its generalization. Researchers could also change the combination of the different variables for future research purposes.

Moreover, this study will provide insight into nurses' minds about their work-family conflict and job pressure in contrast with the health organizations' intrinsic rewards. These insights will help managers re-evaluate their HR policies. They could introduce a more robust and practical intrinsic rewards method in their organization for better productivity and enhanced employee loyalty.

### Study Limitations

This research is conducted in a hospital of a specific province, so it could not be generalized. The geographical area could also be considered as a limitation of that study. Moreover, time and resource limitations are other limitations of this study. It could also be possible that the results could change when the COVID-19 pandemic is over. Moreover, it's a possibility that nurses could not focus on this questionnaire's contents due to their hectic work requirements. This limitation is entirely accurate. Because during the COVID-19 scenario, the work pressure on these nurses is exceptionally high.

Moreover, they also need to look after their home and personal matters during these hectic times. That's why the data collection was an extremely challenging job in this study. However, the questionnaire which is used in this study is authentic, valid, and reliable. It is well written and understandable according to by the nurses according to their job specifications.

## Data Availability Statement

The datasets presented in this article are not readily available because of ethical restrictions. Requests to access the datasets should be directed to the corresponding author.

## Author Contributions

MA, DH, MSar, and MSad: conceptualization. MA, MSar, and MSad: data curation and formal analysis. MA and DH: methodology and supervision. MA and MSar: validation. MA and MSad: writing—original draft. MA, DH, and MSad: writing review and editing. All authors contributed to the article and approved the submitted version.

## Conflict of Interest

The authors declare that the research was conducted in the absence of any commercial or financial relationships that could be construed as a potential conflict of interest.
